# Enterovirus genotype diversity with emergence of coxsackievirus A2 circulating in pediatric patients with acute gastroenteritis in Thailand, 2019–2022

**DOI:** 10.3389/fmicb.2024.1414698

**Published:** 2024-06-03

**Authors:** Zhenfeng Xie, Pattara Khamrin, Nutthawadee Jampanil, Arpaporn Yodmeeklin, Nuthapong Ukarapol, Niwat Maneekarn, Kattareeya Kumthip

**Affiliations:** ^1^Department of Microbiology, Faculty of Medicine, Chiang Mai University, Chiang Mai, Thailand; ^2^Guangxi Colleges and Universities Key Laboratory of Basic Research and Transformation of Cancer Immunity and Infectious Diseases, Youjiang Medical University for Nationalities, Baise, China; ^3^Center of Excellence in Emerging and Re-Emerging Diarrheal Viruses, Chiang Mai University, Chiang Mai, Thailand; ^4^Department of Pediatrics, Faculty of Medicine, Chiang Mai University, Chiang Mai, Thailand

**Keywords:** acute gastroenteritis, coxsackievirus, enterovirus, pediatric patients, Thailand

## Abstract

**Introduction:**

Enteroviruses (EVs) are recognized as potential causative agents of acute gastroenteritis (AGE) in children worldwide. This study aimed to investigate the epidemiology and molecular characteristics of EV infection in children admitted to hospitals with AGE in Chiang Mai, Thailand from 2019 to 2022.

**Methods:**

A total of 1,148 fecal samples collected from patients with AGE were screened for the presence of EV using RT-PCR. The prevalence, co-infection with common diarrheal viruses, and seasonal pattern of EV were examined. The genotypes of EV were identified based on the VP1 sequence and phylogenetic analysis.

**Results:**

The overall prevalence of EV in AGE patients was 8.8% (101/1,148). After the COVID-19 outbreak in 2019, a significant decrease in the EV infection rate and genotype diversity was observed (*p* < 0.05). EV infection alone was observed in 68.3% (69/101) of cases while co-infection with other enteric viruses was 31.7% (32/101). The seasonal pattern of EV infection showed a peak prevalence during the rainy season. EV species A was the most prevalent (37.5%), followed by species B (32.3%), species C (29.2%), and species D (1.0%). Twenty-five genotypes of EV were identified with the most predominant of the coxsackievirus A2 (CV-A2) (13.5%), CV-B2 (7.3%) and CV-A24 (5.2%).

**Conclusion:**

Our data demonstrate a significant decrease in the prevalence and diversity of EV circulating in AGE patients during the COVID-19 pandemic and highlight the emergence of CV-A2 during this study period. These findings contribute to a better understanding of the molecular epidemiology and diversity of EV in patients with AGE and provide useful information for further investigation into the potential association between specific EV genotypes and AGE in future studies.

## 1 Introduction

Acute gastroenteritis (AGE) is a significant cause of illness and death in infants and young children globally, particularly in developing countries (Guarino et al., 2020). Diarrheal diseases accounted for over 500,000 deaths in children under five years old in 2019 (Our World in Data: Diarrheal Diseases https://ourworldindata.org/diarrheal-diseases). Viruses are responsible for more than 70% of AGE cases (Elliott, 2007), with norovirus, rotavirus, astrovirus, adenovirus, and sapovirus being the primary viral pathogens (Bányai et al., 2018). In addition, a number of viral agents identified in the stools of patients with AGE have not yet been confirmed, indicating their potential association with diarrheal disease (Oude Munnink and van der Hoek, 2016). It has also recently been reported that enteroviruses (EVs) are associated with AGE (Rao et al., 2013; Patil et al., 2015).

EVs are non-enveloped, with a positive-sense single-stranded RNA genome (7.5–8.0 kb in length), and about 22–30 nm in diameter. EVs belong to the *Picornaviridae* family and consist of 15 species, including 12 species of EV (EV-A to EV-L) and 3 species of rhinovirus (RV), RV-A to RV-C (Walker et al., 2019). Of these, EV-A to EV-D and RV-A to RV-C infect humans. To date, 116 EV genotypes have been identified within EV species A to D (Simmonds et al., 2020). The partial VP1 sequence has generally been used for identifying the EV genotype because it exhibits a stronger correlation with serotype when compared to other genomic regions (Oberste et al., 1999). EV infection can cause symptoms ranging from mild cold–like conditions to severe diseases affecting various tissues and organs, including the central nervous system, respiratory tract, and the gastrointestinal tract (Rao, 2021).

A number of recent studies have revealed an increasing prevalence of EV in AGE patients worldwide, ranging from 5.3 to 26.3% (Phan et al., 2005; Chitambar et al., 2012; Kumthip et al., 2017; Gosert et al., 2018; Pham et al., 2018; Machado et al., 2020; Perez-Martinez et al., 2021; Gelaw and Liebert, 2022). The studies conducted in Switzerland and Ethiopia demonstrated that EV was one of the three most commonly identified pathogens in patients with AGE (Gosert et al., 2018; Gelaw and Liebert, 2022). Another study in India showed a significant difference in EV infection rates between patients with and without AGE, supporting the potential role of EV infection in AGE (Patil et al., 2015). Although the exact relationship between EV and AGE is not fully understood, it is crucial to continuously monitor and identify these viruses in pediatric patients with AGE. This study describes the epidemiological data and EV genotypes circulating in pediatric patients under five years old diagnosed with AGE in Chiang Mai, Thailand, from 2019 to 2022.

## 2 Materials and methods

### 2.1 Specimen collection

Stool samples were collected from pediatric patients under the age of five years who were admitted with AGE to Maharaj Nakorn Chiang Mai Hospital, Nakorn Ping Hospital, Sanpatong Hospital, Rajavej Chiang Mai Hospital, and Sansai Hospital located in Chiang Mai Province, Thailand. The stool specimens were collected from January 2019 to December 2022. A total of 1,148 stool samples were collected and stored at −20°C until analysis. The study was approved by the Research Ethics Committee of the Faculty of Medicine, Chiang Mai University (MIC-2567-0254).

### 2.2 RNA extraction and reverse transcription

Stool samples were made into 10% fecal suspensions in 1X phosphate-buffered saline (PBS, pH 7.2). The viral genome was extracted from 200 μL of the supernatant of the 10% fecal suspensions using the Geneaid Viral Genomic Extraction Kit (Geneaid, Taiwan) in accordance with the manufacturer’s protocol. Reverse transcription (RT) was performed to synthesize the cDNA using the RevertAid First Strand cDNA Synthesis Kit and a random hexamer primer (Thermo Scientific, United States) in accordance with the manufacturer’s instructions.

### 2.3 Detection of EV and other diarrheal viruses in fecal specimens

For the detection of EV in fecal samples, polymerase chain reaction (PCR) amplification was conducted using primers specific to EV. The sense primer F1 (5′-CAAGCACTTCTGTTTCCCCGG-3′) and antisense primer R1 (5′-ATTGTCACCATAAGCAGCCA-3′) designed to bind to the 5′ untranslated region (UTR) of the EV genome were used to amplify a PCR product of 440 bp (Zoll et al., 1992). The GoTaq DNA Polymerase kit (Promega, United States) was used with the following thermal cycling conditions: initial denaturation at 95°C for 3 min, followed by 35 cycles of denaturation at 95°C for 1 min, annealing at 50°C for 1 min, extension at 72°C for 1 min, and a final extension step at 72°C for 10 min. In addition to the detection of EV, all samples were tested for the presence of other diarrheal viruses as described in previous studies (Drexler et al., 2008; Kapoor et al., 2010; Khamrin et al., 2011). Briefly, 9 pairs of specific primers targeting different types of enteric viruses, including rotavirus A and C, norovirus GI and GII, sapovirus, adenovirus, astrovirus, human parechovirus, and Aichi virus, were used to detect various type of viruses in a single-tube multiplex PCR reaction. The amplification was performed for 35 cycles under the following thermal cycling conditions: denaturation at 94°C for 1 min, annealing at 48°C for 1 min, extension at 72°C for 1 min 15 s, followed by a final extension at 72°C for 10 min (Khamrin et al., 2011). Investigations for the detection of saffold virus and human bocavirus were performed separately. For saffold virus, primers CF188 and CR990 were used to amplify an 800 bp DNA fragment. The thermal cycling conditions for 35 cycles were performed as follows: 94°C for 1 min, 55°C for 1 min, 72°C for 1 min, and final extension at 72°C for 10 min (Drexler et al., 2008). The presence of human bocavirus was determined through nested-PCR using outer and inner primer sets as described previously (Kapoor et al., 2010). Thirty-five cycles of the first-round PCR were as follows: 94°C for 3 min prior to denature at 94°C for 1 min, annealing at 55°C for 1 min, and extension at 72°C for 1 min, followed by a final extension at 72°C for 10 min. Similar conditions were used for the second round PCR with the exception of an annealing temperature at 58°C for 1 min. After completion of PCR amplification, the PCR products were analyzed using 1.5% agarose gel electrophoresis.

### 2.4 Genotyping of EV

Fecal samples that tested positive for EV were subjected to genotyping by amplifying the partial VP1 region. Nested-PCR amplification was performed using outer primers 224 (5′-GCIAT GYTIGGIACICAYRT-3′) and 222 (5′-CICCIG GIGGIAY RWACAT-3′), followed by inner primers AN89 (5′-CCAGCACTGACAG CAGYNGARAYNGG-3′) and AN88 (5′-TACTGGACCACCTG GNGGNAYRWACAT-3′), as described previously (Nix et al., 2006). The PCR reagents and thermal cycling conditions were consistent with the EV detection protocol with the exception of the annealing temperature in the first and second round PCR were set at 42°C and 55°C, respectively. Additionally, the number of amplification cycles in the second round PCR was extended to 40 cycles. The amplicon sizes of the first and the second-round PCR were 993 and 350 base pairs, respectively. The PCR products were purified using the GenepHlow gel/PCR Kit (Geneaid, Taiwan) and then subjected to direct sequencing using the BigDye Terminator v3.1 Cycle Sequencing Kit (Thermo Scientific, United States). The sequencing process was conducted by the 1st BASE laboratories (Apical Scientific Sdn. Bhd, Malaysia) operating on the ABI PRISM 3730xl Genetic Analyzer (Applied Biosystems, United States). The sequences obtained were analyzed to initially define the genotype of the EV by comparing with those of the reference strains available in the GenBank database using the Basic Local Alignment Search Tool (BLAST) server^1^ and the Enterovirus Genotyping Tool version 1.0.^2^

### 2.5 Phylogenetic analysis

To construct a phylogenetic tree, we included the prototype strains of each EV genotype and three EV strains in the GenBank database that exhibited the highest nucleotide sequence identity with each of our EV strains. Sequences of the EV strains that are commonly incorporated into the phylogenetic analyses by other previous studies were also included. Multiple sequence alignments of the partial VP1 sequences of EV were conducted using MEGA X software and the ClustalW program. Subsequently, maximum likelihood (ML) phylogenetic trees for EV species A, B, C, and D were constructed using the IQ-TREE (v2.2.2.6), employing specific nucleotide substitution best-fit models. Specifically, the TIM2e + I + G4 model was used for species A and B, while the TIM2 + F + G4 and HKY + F + G4 models were applied in the case of species C and D. ModelFinder was used to determine the selection of the nucleotide substitution best-fit models based on the Bayesian information criterion (BIC) (Kalyaanamoorthy et al., 2017). To assess the robustness of the phylogenetic trees, an ultrafast bootstrap (UFBoot) with 5000 replicates and a Shimodaira-Hasegawa-like approximate likelihood-ratio test (SH-aLRT) with 1000 replicates was performed.

### 2.6 The nucleotide sequence accession numbers of EV

Nucleotide sequences of partial VP1 region of EV strains analyzed in this study are available in the GenBank database under the accession numbers OR500115–OR500210.

### 2.7 Statistical analysis

The statistical analyses were performed using SPSS 27.0 software. Categorical variables were analyzed using the chi-square (*χ*^2^) test, and a *p*-value less than 0.05 was considered statistically significant.

## 3 Results

### 3.1 Prevalence of EV infection and co-infection in AGE patients

The prevalence of EV infection and co-infection in children with AGE from 2019 to 2022 is summarized in Table 1. From a total 1,148 samples tested, 101 (8.8%) were positive for EV. The prevalence of EV infection varied year by year, ranging from 5.4 to 11.7%. The highest detection rate was observed in 2019 with a prevalence of 11.7%, while the lowest infection rate was found in 2021 with a prevalence of 5.4%. It was noteworthy that the prevalence of EV infection decreased continuously from 2020 (9.6%), 2021 (5.4%, *p* = 0.006) and 2022 (5.5%, *p* = 0.008) after the COVID-19 pandemic. The prevalence of sole EV infection was found in 69 out of 101 AGE cases (68.3%), whereas co-infection of EV with other diarrheal viruses was observed in 32 out of 101 (31.7%). Among the 32 co-infected cases, 27 were co-infected with a single other diarrheal virus. These included adenovirus (3.1%, 1 case), human bocavirus (15.6%, 5 cases), norovirus (40.6%, 13 cases), rotavirus (9.4%, 3 cases), sapovirus (9.4%, 3 cases), saffold virus (3.1%, 1 cases), and human astrovirus (3.1%, 1 case) (Table 1). In addition, 4 cases (12.5%) were co-infected with two diarrheal viruses (HBoV + SaV, 2 cases; NoV + RV, 1case; HBoV + NoV, 1 case), and one (3.1%) was co-infected with three enteric viruses (HBoV+NoV + RV).

**Table 1:** Prevalence of EV single infection and co-infection in children with acute gastroenteritis from 2019 to 2022.

Year	AGE cases	EV-positive cases *n* (%)	*χ*^2^ value	*p*-value	Number and patterns of EV co-infection								
					AdV^b^	HBoV^c^	NoV^d^	RV^e^	SaV^f^	SAFV^g^	HAstV^h^	2 viruses	3 viruses
2019	513	60 (11.7)	—	—	1	3	7	1	1	1	—	2 (c + f)	1 (c + d + e)
2020	156	15 (9.6)	0.52	0.471	—	—	4	1	—	—	1	1 (d + e) 1(c + d)	-
2021	242	13 (5.4)	7.529	0.006^a^	-	2	2	1	1	—	—	—	—
2022	237	13 (5.5)	7.117	0.008^a^	—	—	—	—	1	—	—	—	—
Total	1148	101 (8.8)	—	—	1	5	13	3	3	1	1	4	1

a A Pearson-chi-square test was performed to examine the statistical differences in EV positive rates between the post-COVID-19 period of 2020–2022 and the pre-COVID-19 period of 2019.

b Adenovirus (AdV).

c Human bocavirus (HBoV).

d Norovirus (NoV).

e Rotavirus (RV).

f Sapovirus (SaV).

g Saffold virus (SAFV).

h Human astrovirus (HAstV).

### 3.2 Prevalence of EV infection in different age groups

**Figure 1:**
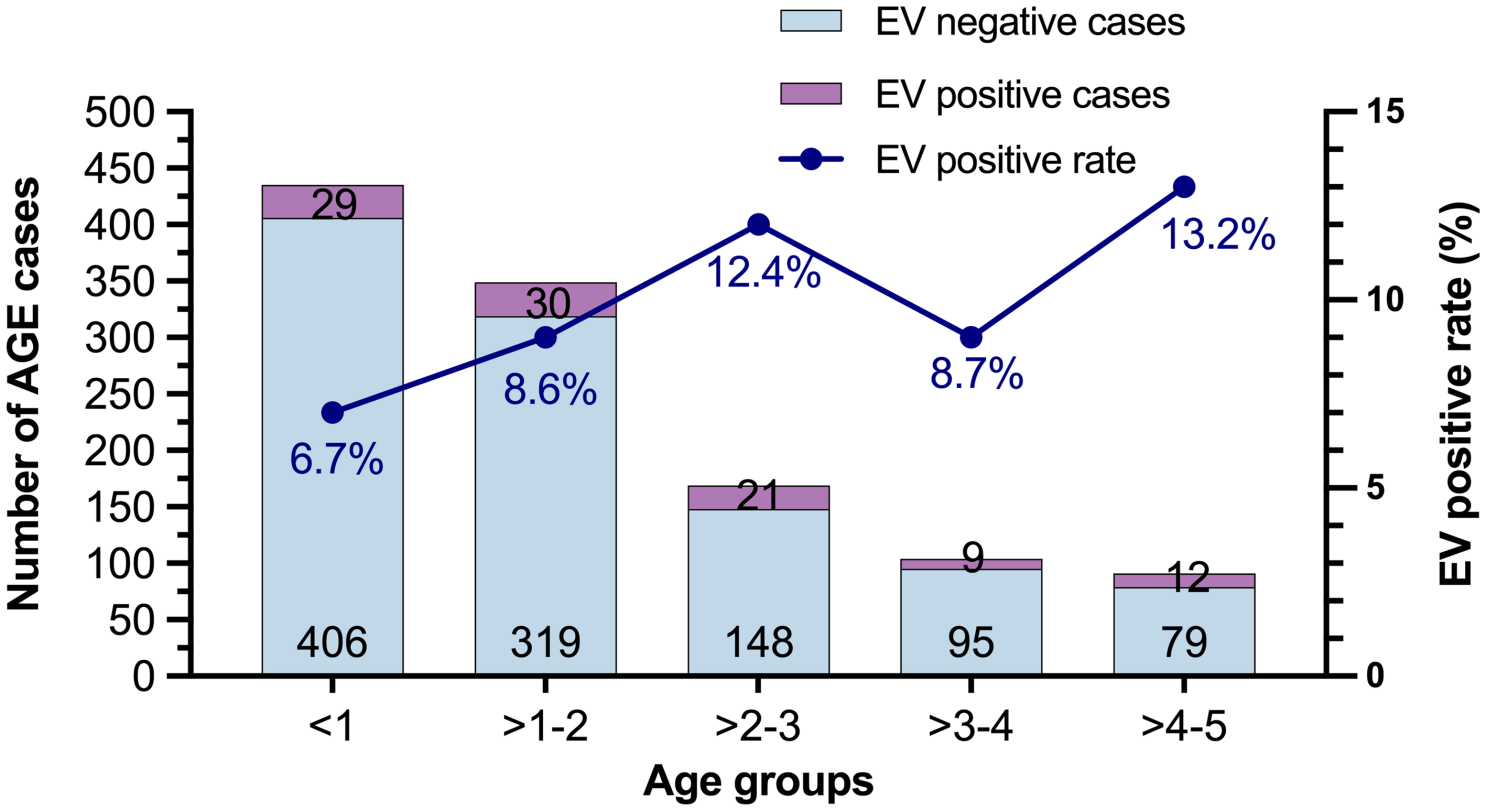
The prevalence of EV infection in different age groups from 2019 to 2022. The number of EV-negative and EV-positive cases are labeled at the bottom and top of the bars, respectively.

The prevalence of EV infection in children with different age groups is shown in Figure 1. The highest prevalence of EV infection was observed in children in the age group between 4 and 5 years (13.2%), followed by children in age group of >2–3 years (12.4%), >3–4 years (8.7%), >1–2 years (8.6%), and <1 year (6.7%). However, there was no statistically significant difference between the prevalence in these age groups (*χ*^2^ = 7.440, *p* = 0.114).

### 3.3 Distribution of EV species and genotypes

The distribution of EV species and genotypes identified in this study is shown in Table 2. Out of 101 EV-positive samples, the genotypes of 96 were successfully defined based on the VP1 sequence while 5 samples of the VP1 gene had failed to be amplified. Among the 96 cases, 36 were EV species A (37.5%), 31 were species B (32.3%), 28 were species C (29.2%), and 1 was species D (1.0%). Among 25 different EV genotypes, coxsackievirus A2 (CV-A2), poliovirus 3 (PV3), and CV-B2 were the most predominant genotypes detected in this study. In the case of EV species A, eight distinct genotypes were identified in this study, including coxsackievirus A2 (CV-A2), CV-A4, CV-A5, CV-A6, CV-A8, CV-A10, CV-A16, and EV-A71. CV-A2 was the most common genotype, accounting for 13.5% (13/96). Among 13 CV-A2 infected cases, co-infection with other gastroenteritis viruses was observed in 7 cases. In EV species B, 10 different genotypes including CV-A9, CV-B2, CV-B3, Echovirus 6 (E6), E11, E14, E16, E20, E21, and E25 were identified. Of these, CV-B2 was the most prevalent genotype (7.3%, 7/96). Furthermore, six genotypes within species C, including poliovirus 1 (PV1), PV3, CV-A1, CV-A24, EV-C96, and EV-C99. were detected. The PV3 was the most predominant genotype identified within species C, accounting for 12.5% (12/96). A single species D detected in this study was EV-D68, which accounted for 1.0% (1/96). Notably, the diversity of EV genotypes showed a remarkable decrease during the COVID-19 pandemic. In 2019, 21 different genotypes of EV were identified whereas only 5, 9, and 10 genotypes were detected in 2020, 2021, and 2022, respectively (Table 2).

**Table 2:** Distribution of EV species, genotypes, and co-infection cases identified in acute gastroenteritis patients from 2019 to 2022.

Year	Species A (%)								Species B (%)										Species C (%)						Species D (%)	^a^No. of strains
	CV-A2	CV-A4	CV-A5	CV-A6	CV-A8	CV-A10	CV-A16	EV-A71	CV-A9	CV-B2	CV-B3	E6	E11	E14	E16	E20	E21	E25	PV1	PV3	CV-A1	CV-A24	EV-C96	EV-C99	EV-D68	
2019	10 (17.9)	5 (8.9)	1 (1.8)	1 (1.8)	3 (5.4)	1 (1.8)	2 (3.6)	—	2 (3.6)	7 (12.5)	1 (1.8)	—	3 (5.4)	1 (1.8)	3 (5.4)	2 (3.6)	2 (7.1)	—	2 (3.6)	2 (3.6)	3 (5.4)	3 (5.4)	1 (1.8)	—	1 (1.8)	56
2020	1 (7.1)	—	—	—	—	—	—	3 (21.4)	—	—	—	4 (28.6)	—	—	—	—	—	—	—	5 (35.7)	—	—	—	1 (7.1)	—	14
2021	1 (7.1)	—	—	—	—	2 (15.4)	1 (7.7)	—	1 (7.7)	—	—	—	—	—	—	—	1 (7.7)	—	1 (7.7)	4 (30.8)	—	1 (7.7)	1 (7.7)	—	—	13
2022	1 (7.7)	—	1 (7.7)	2 (15.4)	1 (7.7)	1 (7.7)	—	—	2 (15.4)	—	—	—	—	—	—	—	—	2 (15.4)	1 (7.7)	1 (7.7)	—	1 (7.7)	—	—	—	13
Total	13 (13.5)	5 (5.2)	2 (2.1)	3 (3.1)	4 (4.2)	4 (7.1)	3 (3.1)	3 (3.1)	5 (5.2)	7 (7.3)	1 (1)	4 (4.2)	3 (3.1)	1 (1)	3 (3.1)	2 (2.1)	3 (3.1)	2 (2.1%)	4 (4.2)	12 (12.5)	3 (3.1)	5 (5.2)	2 (2.1)	1 (1)	1 (1)	96
^b^CI	7	—	—	—	—	1	2	—	3	2	—	2	1	1	—	1	1	1	—	4	—	3	1	1	—	31

a Number of EV strains as typed by VP1 sequence.

b CI: Co-infection cases identified in each genotype.

### 3.4 Seasonal distribution of EV infection

**Figure 2:**
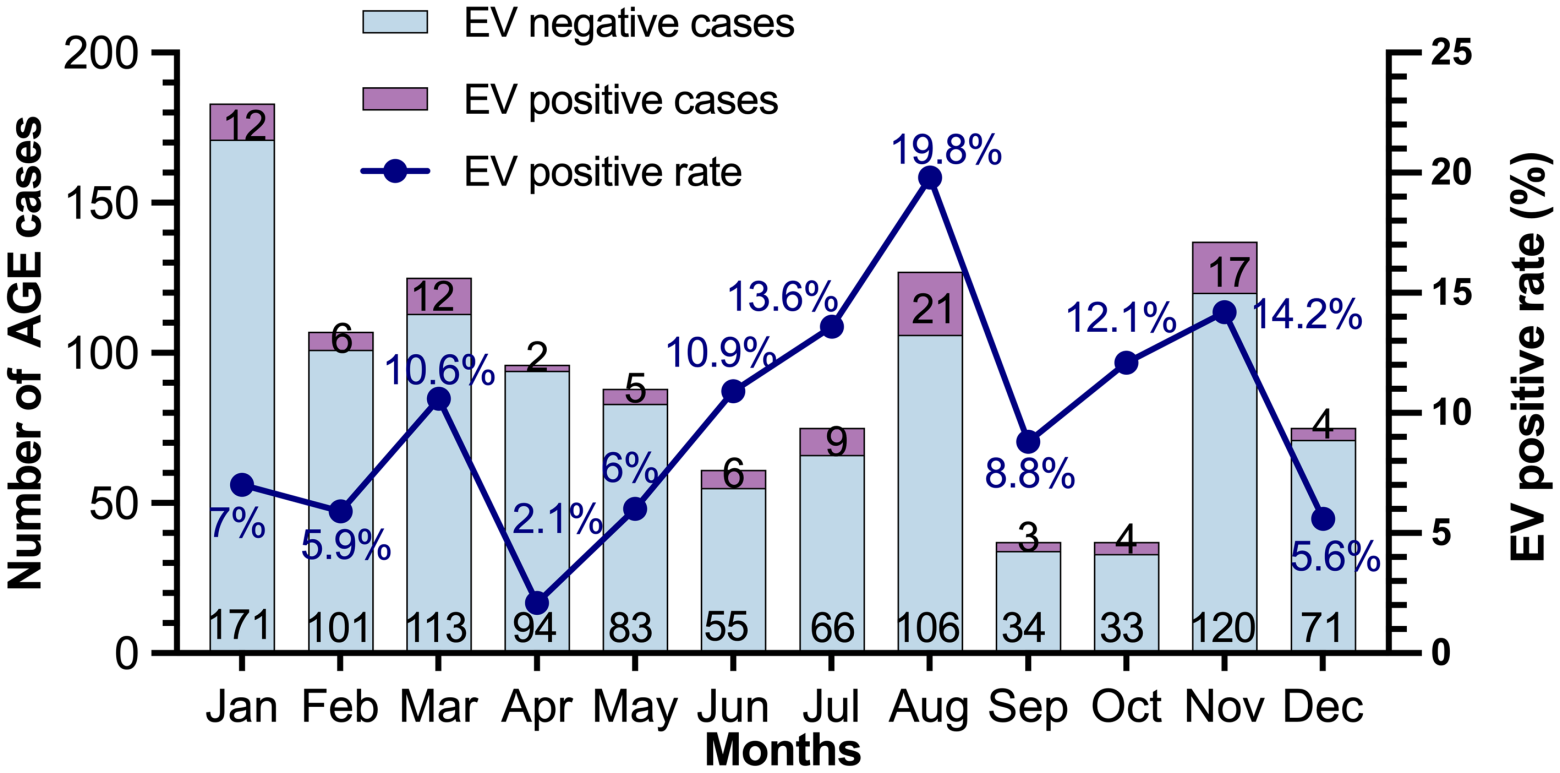
Seasonal distribution of EV in patients with acute gastroenteritis during 2019–2022. The number of EV-negative and EV-positive cases are indicated at the bottom and top of each bar, respectively.

The seasonal distribution of EV infection in pediatric patients with AGE is shown in Figure 2. EV infection was generally detected throughout the year with a high prevalence during the rainy season in Thailand (July–October). In this study, the EV infection rate peaked in July (13.6%) and August (19.8%), high levels of 12.1 and 14.2% also recurring in October and November, respectively.

### 3.5 Phylogenetic analysis of EV strains

**Figure 3:**
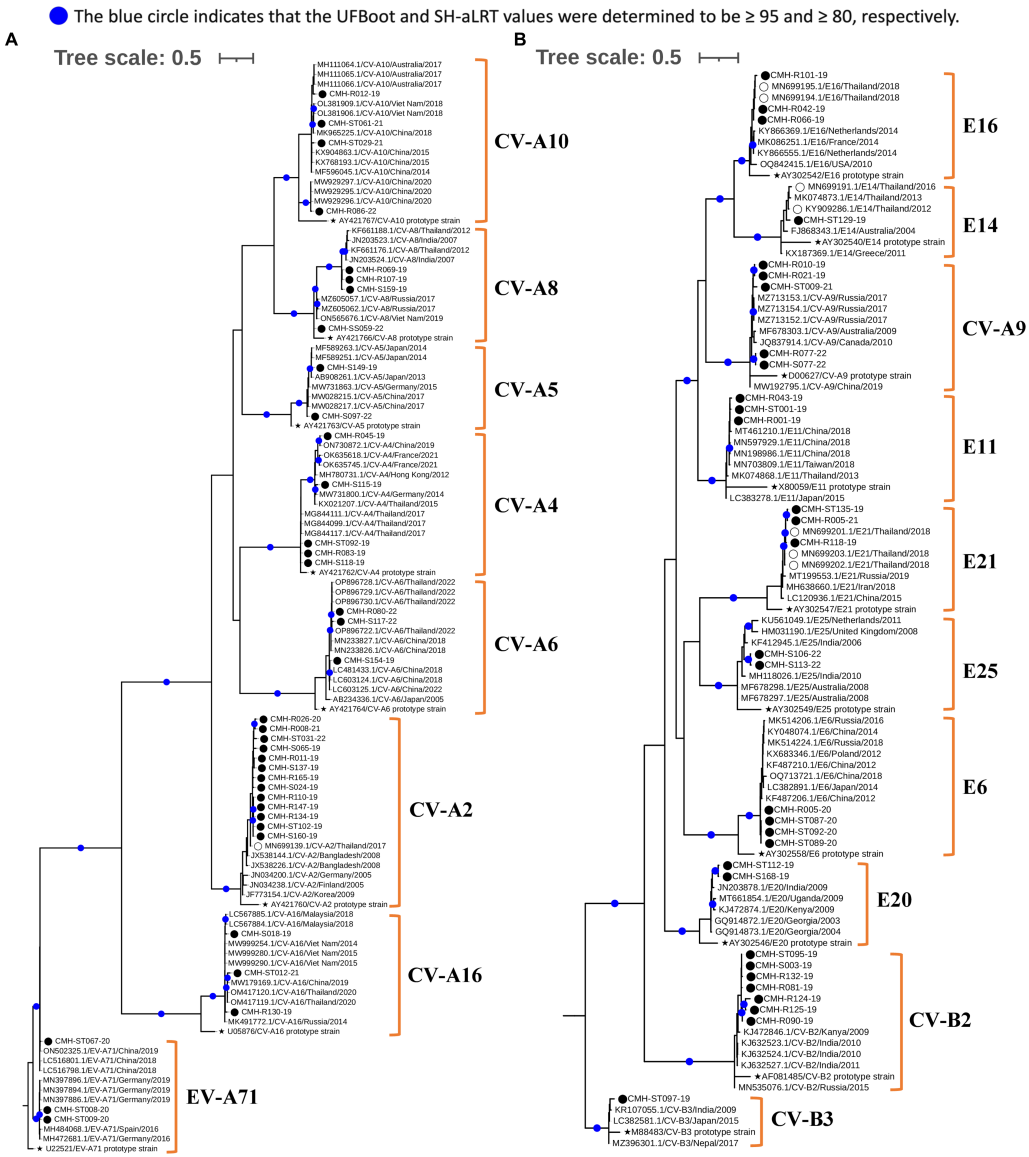
Phylogenetic analysis of EV species A **(A)** and B **(B)**. Phylogenetic trees were constructed using IQ-TREE (v2.2.2.6) based on partial VP1 nucleotide sequences employing the maximum likelihood method. Nodes with UFBoot and SH-aLRT support values ≥95 and 80, respectively, are indicated by blue circles. EV strains identified in this study are denoted by black circles (⬤), a star symbol represents the prototype reference strains of each genotype (★), and reference strains detected previously in Chiang Mai, Thailand are indicated by empty circles (◯).

**Figure 4:**
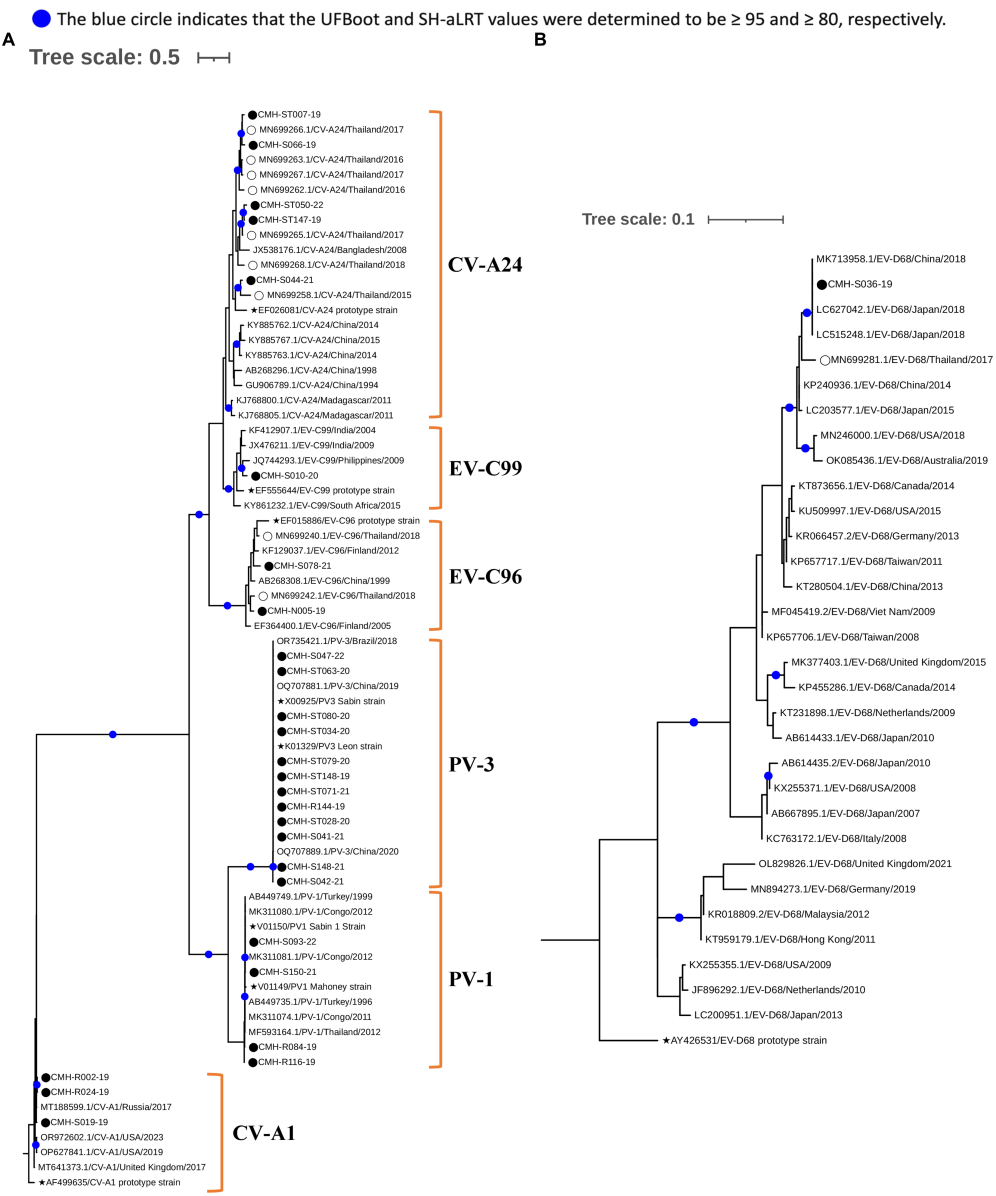
Phylogenetic analysis of EV species C **(A)** and D **(B)**. Phylogenetic trees were constructed using IQ-TREE (v2.2.2.6) based on partial VP1 nucleotide sequences employing the maximum likelihood method. Nodes with UFBoot and SH-aLRT support values ≥95 and 80, respectively, are indicated by blue circles. EV strains identified in this study are denoted by black circles (⬤), a star symbol represents prototype reference strains of each genotype (★), and reference strains detected previously in Chiang Mai, Thailand are indicated by empty circles (◯).

To investigate genetic relationships between the 96 EV strains detected in this study and the previously reported EV reference strains, the phylogenetic tree of each EV species (A–D) was constructed as shown in Figures 3, 4. The overall results demonstrated that all EV strains detected in this study clustered closely together with their corresponding genotype reference strains. Each of the 25 EV genotypes formed distinct branches together with the corresponding reference strains, confirming the reliability of genotyping results of EV strains as determined by BLAST and the Enterovirus Genotyping Tool. Almost all EV strains shared more than 90% nucleotide sequence identity with their corresponding reference genotypes, with the exception of CV-A24, EV-C96, and EV-C99 which had lower sequence identity to their reference sequences (79.9–89.5%).

Regarding phylogenetic relationships, the majority of EV strains in this study exhibited close genetic similarity to the EV reference strains reported from Asia whereas a minority of our EV strains showed a close genetic relationship with EV reference strains reported from Europe, Australia, and the United States. Focusing on species A, most of our strains were most closely related to the reference strains previously reported from several countries in Asia, including Bangladesh, China, Hong Kong, India, Japan, Korea, Malaysia, Thailand, and Vietnam. Some EV strains of CV-A4, CV-A16, and EV-A71 exhibited a close nucleotide sequence similarity to the reference strains reported from Australia, Germany, and Russia (Figure 3A). All CV-A9 and CV-B2 strains in species B were closely related to the reference strains from Russia and Kanya, respectively. The other EV strains in species B were more closely related to the reference strains previously detected in Thailand, China, and India (Figure 3B). For species C, all EV strains showed a close genetic relationship with reference strains from Thailand and India, except for the CV-A1 and EV-C99 strains, which were closely related to the reference strains reported from Russia and Philippines, respectively. It’s noteworthy that all PV1 and PV3 identified in this study were polio vaccine-associated strains sharing 98–100% nucleotide sequence identity with PV1 and PV3 Sabin vaccine strains (Figure 4A). The single EV-D68 strain detected in this study showed a close genetic similarity with reference strains from China and Japan (Figure 4B).

## 4 Discussion

Before the COVID-19 pandemic, the prevalence of EV infection in AGE patients varied widely, ranging from 5.3 to 26.3% in different countries. The lowest EV prevalence was reported in Switzerland (Gosert et al., 2018), while the highest EV infection rate was reported from Brazil (Machado et al., 2020). In Thailand, the prevalence of EV infection in children with AGE was reported at 5.8 and 8.9% during 2010–2014 and 2015–2018, respectively (Kumthip et al., 2017; Rojjanadumrongkul et al., 2020). Concurrently, two studies conducted in Japan reported an EV prevalence in AGE patients of 6.1% during 2009–2013 and of 6.5% during 2014–2016 (Thongprachum et al., 2015; Pham et al., 2018). The higher prevalence of EV infection detected in AGE patients in Asian countries was reported from India at 13.7 and 19.1% during 2004–2009 and 2008–2013, respectively (Rao et al., 2013; Patil et al., 2015). An increasing trend in EV prevalence in AGE patients was observed across different Asian countries before the COVID-19 outbreak, beginning at the end of 2019. Notably, this study revealed an increased EV prevalence in 2019 (11.7%) compared to the overall prevalence detected in Thailand during 2015–2018. However, significant decreases in the number of AGE cases, EV prevalence, and genotype diversity were observed during the COVID-19 pandemic (2021–2022) compared to the pre-COVID-19 period (2019). The decline in the number of AGE cases and EV prevalence might be due to several factors, including changes in people’s behavior (increased hygiene, social distancing, and mask wearing), disruption of healthcare access (due to COVID lockdowns, quarantine measures, and the overwhelming of healthcare systems by COVID-19 cases), and the policies implemented to control the spread of COVID-19. In addition, the possible scenario where AGE cases were ignored or underreported while authorities were focused on the COVID-19 pandemic cannot be ruled out. A number of similar studies conducted in clinical settings during the COVID-19 pandemic indicated that the infection control measures, including the enforcement of social distancing protocols, COVID lockdowns in the outbreak areas, childcare and school closures, and face mask wearing, resulted in a significant decrease in the incidence of several enteric pathogens such as enterovirus, norovirus, rotavirus and adenovirus (Bruggink et al., 2021; Douglas et al., 2021; Zhang et al., 2022; Pham et al., 2023). Similar findings of the decrease in EV prevalence have been reported in Australia and America in 2020 (Bruggink et al., 2021; Kies et al., 2021). In addition, a decline in the prevalence of EV infection and other enteric viruses, such as rotavirus and adenovirus, during the same period has also been reported in China (Zhang et al., 2022). These findings suggest a potential influence of the control measures for the COVID-19 pandemic on a decline in the prevalence of EV infection and genotype diversity in pediatric patients with AGE.

Among 101 EV-positive cases, co-infection with other common diarrheal viruses was found in 31.7% (32 out of 101) of cases, a finding similar to the studies conducted in Switzerland (33%) (Gosert et al., 2018) and Spain (39.7%) (Perez-Martinez et al., 2021). Notably, single infection by EV was as high as 68.3% in this study, suggesting a causative effect in AGE patients. Norovirus was identified as the predominant co-infection agent, accounting for 50% (16 out of 32) of co-infection cases. It should be noted that norovirus was more frequently detected in patients with AGE in Thailand during the same period (Jampanil et al., 2021; Wei et al., 2021; Phengma et al., 2022; Hogben et al., 2023; Yodmeeklin et al., 2023). Similarly, adenovirus was the most predominant virus detected in AGE patients in Spain from 2013 to 2017, and it was found to be the most common co-infection virus detected in EV-positive cases (Perez-Martinez et al., 2021). Co-infection is frequently observed among AGE patients although the rates of co-infection vary depending on the country and the number of pathogens detected (Makimaa et al., 2020). Higher rate of co-infection seems to be observed frequently in the studies conducted in low-income countries (Tandukar et al., 2019). Furthermore, the presence of co-infection may raise concerns about the risk and severity of AGE. Studies from the Netherlands (Pijnacker et al., 2019), Japan (Hikita et al., 2023), and Nepal (Tandukar et al., 2019) demonstrated that co-infection did not increase the risk or severity of AGE. Conversely, a study conducted in Israel indicated that co-infection was associated with more severe clinical symptoms in children under five years old (Hazan et al., 2024). Although the impact of co-infection on the severity of AGE remains unclear, the detection of viral load in stool samples using real-time PCR could be useful in assessing the contribution of individual viruses to the severity of disease as well as virus-virus interaction in co-infection cases (De Grazia et al., 2020).

In this study, the seasonal distribution of EV-positive cases demonstrated an increase in the prevalence of EV infection during the rainy season, which typically falls between July and October in Chiang Mai, Thailand. The combination of rainfall and warm temperatures during this period creates an environment that facilitates the transmission of EVs, as they are predominantly transmitted through the fecal-oral route (Lipp et al., 2001).

Several studies conducted in Brazil, India, and Japan have demonstrated that EV species B was the most frequently detected in patients with AGE and accounted for more than 50% of all EV-infected cases (Patil et al., 2015; Pham et al., 2018; Machado et al., 2020). However, the present study found that EV species A became the most predominant species identified in AGE patients. A marked increase in the detection rate of CV-A2 within the species A in 2019 may help to explain this phenomenon. In addition, several EV genotypes in species A, including CV-A4, CV-A6, CV-A10, and EV-A71 were reported as the most common agents for hand-foot-mouth-disease (HFMD) patients in Thailand during the same study period as reported by another team of investigators (Thammasonthijarern et al., 2021). The predominance of these EV genotypes circulating in Thailand are likely contributing to the higher prevalence of EV infection caused by species A viruses in AGE patients. However, to elucidate this association, further studies need to be carried out.

A wide range of EV genotypes have been identified in patients with AGE (Rao, 2021). In Brazil and India, specific echovirus genotypes E7, E9, E11, and E13 have been commonly found in patients with AGE (Rao et al., 2013; Machado et al., 2020). Additionally, E6 and E11 have been frequently detected in the AGE outbreaks in Japan and India, respectively (Patel et al., 1985; Abe et al., 2000). Regarding coxsackievirus, several genotypes, including CV-A4, CV-A5, CV-A24, and CV-B5, have been frequently detected in AGE patients in Japan and Thailand (Kumthip et al., 2017; Pham et al., 2018; Rojjanadumrongkul et al., 2020). The present study found the predominant genotypes reported in the previous studies, specifically, E6, E11, CV-A4, CV-A5, and CV-A24 are still accounted for 19.8% (19/96) of EV infection cases, while CV-A2 and PV3, the most prevalent genotypes detected in this study were responsible for 26.0% (25/96) of cases. Even excluding co-infection cases with CV-A2 infection, this was still the predominant genotype identified in AGE patients, suggesting its potential role in diarrheal disease. In addition, the PV vaccine strains detected in the present study were PV1 (25%, 4/16) and PV3 (75%,12/16). Among these, 93.8% (15/16) of PV-positive cases were detected in patients within the age recommended (neonates to 6 months old) for bivalent oral poliovirus vaccine (OPV) administration. In Thailand, three doses of bivalent oral polio vaccine (OPV), which include PV1 and PV3 strains, are routinely administrated to the neonates at 2, 4, and 6 months after birth. The first and second booster are given at 18 months old and between 4 and 6 years old, respectively (Garg et al., 2018). Only 25% (4/16) of PV-positive cases were found to be co-infected with other common gastroenteritis viruses while the majority (75%) were solely PV infected. These findings suggest that the administration of bivalent OPV may contribute to gastrointestinal symptoms in children who received OPV. However, two limitations of this study are the absence of a control group and the detailed clinical information of the patients, making it challenge to determine the association between EV genotype, specifically CV-A2 and PV, and AGE.

EV-D68 is typically associated with respiratory symptoms and acute flaccid myelitis and is primarily transmitted through the respiratory tract (Aliabadi et al., 2016; Cabrerizo et al., 2017; Chong et al., 2018). It is relatively rare to detect EV-D68 in stool samples. A case study reported in Japan in 2015 revealed sole detection of EV-D68 in the fecal sample of a patient with AGE was made through multiplex-PCR screening for several diarrheal viruses (Pham et al., 2017). Interestingly, EV-D68 was also detected in our previous study conducted in Chiang Mai, Thailand in 2017 (Rojjanadumrongkul et al., 2020) and observed again in the present study, with EV-D68 being the only virus detected among several gastroenteritis viruses. However, clinical records of the patient and viability of infectious virion isolated from stool sample are not available, further studies are needed to elucidate the role of EV-D68 in gastrointestinal disease.

The phylogenetic analyses conducted in this study contribute to a better understanding of phylogenetic relationships and genetic diversity of EVs in Chiang Mai, Thailand. A close genetic relationship between EV strains detected in Thailand and neighboring Asian countries suggest the circulation of highly similar EV strains in the same geographical regions. Given the continuous travelling of people between Thailand and neighboring Asian countries, especially in the case of Chiang Mai which is a popular tourist destination attracting significant numbers of travelers from Asian countries, it could be one of the reasons why most EV strains detected in Chiang Mai exhibited a close genetic relationship with the reference strains from Asia. Nevertheless, some EV strains (CV-A2, CV-A8, CV-A24, CV-B2, and E25) formed their own distinct subcluster in the phylogeny, suggesting the genetic divergence of these EV genotypes from the strains circulating in other countries. Future studies should aim to perform more detailed analyses, relying on full-length VP1 or whole genome sequences, to investigate these genetically divergent EV strains.

This study reveals a significant decrease in the prevalence and genotype diversity of EV circulation in pediatric patients hospitalized with AGE in Chiang Mai Thailand from 2019 to 2022 during the COVID-19 pandemic and highlights the emergence of CV-A2 during this study period. These findings enhance our understanding of the molecular epidemiology of EV in Thailand and facilitate the carrying out of future studies investigating potential associations between specific EV genotypes and AGE. However, there are some limitations of this study that need to be considered. Firstly, the details of clinical presentations including symptoms and severity of the disease of the patients enrolled in this study were not available making it challenging to evaluate the actual role of EV in AGE. Secondly, full-length VP1 sequencing should be used for EV classification, however, due to high diversity of EV genome among different EV genotypes and difficulty in amplifying the full-length VP1 gene, the partial VP1 sequence was used to identify the EV genotypes in this study.

## Data availability statement

The datasets presented in this study can be found in online repositories. The names of the repository/repositories and accession number(s) can be found at: https://www.ncbi.nlm.nih.gov/genbank/, OR500115–OR500210.

## Ethics statement

The studies involving humans were approved by the Research Ethics Committee of Faculty of Medicine, Chiang Mai University. The studies were conducted in accordance with the local legislation and institutional requirements. The human samples used in this study were acquired from a by-product of routine care or industry. Written informed consent for participation was not required from the participants or the participants’ legal guardians/next of kin in accordance with the national legislation and institutional requirements.

## Author contributions

ZX: Data curation, Formal analysis, Investigation, Methodology, Software, Visualization, Writing – original draft. PK: Conceptualization, Funding acquisition, Methodology, Supervision, Validation, Visualization, Writing – review & editing. NJ: Investigation, Writing – review & editing. AY: Investigation, Writing – review & editing. NU: Resources, Visualization, Writing – review & editing. NM: Conceptualization, Funding acquisition, Methodology, Resources, Supervision, Validation, Visualization, Writing – review & editing. KK: Conceptualization, Formal analysis, Funding acquisition, Methodology, Supervision, Validation, Visualization, Writing – review & editing.

## Footnotes

^1^https://blast.ncbi.nlm.nih.gov/Blast.cgi

^2^https://www.rivm.nl/mpf/typingtool/enterovirus/
